# Does the presence of a friend reduce cardiovascular response to stress even over a screen?

**DOI:** 10.1371/journal.pone.0314562

**Published:** 2024-12-04

**Authors:** Ayumi Kambara, Hisashi Mitsuishi, Yuki Harada

**Affiliations:** 1 Faculty of Humanities, Kyoto University of Advanced Science, Kyoto, Japan; 2 Faculty of Health and Medical Science, Kyoto University of Advanced Science, Kyoto, Japan; Public Library of Science, UNITED KINGDOM OF GREAT BRITAIN AND NORTHERN IRELAND

## Abstract

Although meeting close friends through video calls has become common, less is known about its stress-buffering effect. This study aims to examine whether cardiovascular responses to stress are decreased when the presence of a friend on a monitor compared to the presence of a friend in person or alone. Forty-six undergraduate students completed a stress task while in the room with a friend (1) in person (in-person condition), (2) on Zoom (online condition), or (3) alone (alone condition). Blood pressure and heart rate were monitored throughout the experiment. Analyses revealed that diastolic blood pressure after the stress was recovered closer to the pre-stress state under in-person and online conditions than alone condition. However, the study observed no differences across groups regarding self-reported affect. In summary, this result suggests that even the online presence of a friend, as well as in-person friends, may help recover blood pressure to the original state without one’s awareness.

## Introduction

Coronavirus disease 2019 (COVID-19) has significantly changed our way of life. Online communication through various platforms, such as Zoom, Skype, or Line, has become common [1]. These onscreen meetings are not only common in work-related scenarios but also for private relationships such as those between friends, romantic partners, and family members. For instance, virtual dates and online communication with partners became more prevalent during the lockdowns [2, 3]. These phenomena indicate the compelling need of people to spend time with close others.

What are the benefits of spending time with close others? Scholars have long reported that social ties reduce the risk of coronary heart disease [4, 5]. As a reason for this, the social support–reactivity hypothesis posits that the presence of close others buffers cardiovascular responses to acute stressors. It is believed that this buffering effect, repeated daily, reduces cardiovascular disease incidence [6, 7]. Therefore, feeling close to others during stressful periods is vital for our health.

Numerous laboratory studies have investigated the effects of close others in buffering acute stress reactions to confirm the social support–reactivity hypothesis. In such experiments, participants were asked to perform stressful tasks (e.g., calculating, speech), and the presence of their close friends (or pets) was experimentally manipulated. They have taken two characteristic forms, namely, passive and active support paradigms [6]. An interesting aspect of the passive support paradigm is that it examined the effect of the *mere presence* of close others on cardiovascular reactivity to stressors. That is, their close others in those experiments were just sitting there because direct communication was prohibited. Those studies have consistently demonstrated this stress-buffering effect of the mere presence of a friend. Despite certain exceptions, this effect has become firmly accepted as a reliable fact in cardiovascular psychophysiology [8–16].

If such a stress-buffering effect occurs simply by feeling the mere presence of a friend, then physical proximity to a friend may not be necessary for this effect to occur. In other words, this effect might also be observed with the online presence of a friend. However, the effects of the presence of a close other have predominantly been examined in face to face scenarios despite the widespread prevalence of online communication. To the best of our knowledge, no study has yet explored these effects on online scenarios.

There are a few studies that have examined the influence of another person through a screen on physiological indicators. For example, one study reported that being seen by others, even via a monitor, elicits the same physiological responses in people as being seen by others in person [17]. Another study reported that eating with a friend via a monitor rather than eating alone enhanced the autonomic nervous system activity during the meal [18]. However, Hetherington et al. [18] did not solely measure the influence of a friend’s presence, because their setup allowed conversation with the friend through the screen. Nevertheless, both studies highlight the potential for people to feel the presence of others even in online situations.

As previously mentioned, lacking a connection with close others for a long period is detrimental to health because the presence of close others buffers cardiovascular responses to acute stressors, leading to a reduction in cardiovascular disease. However, people are not always in environments or situations that enable them to see close others physically. If this is the case, then whether this stress-buffering effect of friend’s mere presence on cardiovascular response can also be achieved via screen warrants an investigation.

The current study aims to examine the effect of the online presence of a friend on cardiovascular responses to stress compared to in-person presence or alone. Cardiovascular responses to a stressor are characterized by an increase in activity during a stressful event or task followed by a decline in activity after the event concludes [19]. As aforementioned, studies examining the effects of close others on cardiovascular responses have found that their presence reduces cardiovascular reactivity to stress and facilitates a more rapid recovery to baseline [9]. Therefore, in this experiment, participants undergo a stress task, and their cardiovascular activity was measured at pre-stress, during, and post- stress. If participants can feel the presence of their friends online, the degree of increase in cardiovascular responses during stress would be smaller, and recovery to the pre-stress state more quickly when they are with a friend online or in person compared to being alone.

Toward this end, in terms of indicators of cardiovascular response to stress, the study measured heart rate (HR), autonomic nervous system (low frequency/high frequency [LF/HF] ratio, HF, and root mean square of successive differences [RMSSD]), and blood pressure (BP). The autonomic nervous system is one of the major neural pathways activated by stress. In stressful scenarios, it is activated without the normal counteraction of the parasympathetic nervous system. The LF/HF ratio is an index of sympathetic nerve activity, while the HF and RMSSD are indices of parasympathetic nerve activity. Diastolic BP (DBP) reflects changes in BP associated with mentally stressful situations beyond one’s control [20, 21]

In addition, the study measured self-reported emotions to explore their association with cardiovascular responses. It has been discussed whether the presence of a friend directly influences the cardiovascular system without subjective emotional changes or is mediated by subjective emotions. Many studies have reported that the presence of a friend directly affects cardiovascular responses without changing subjective affect [6, 8, 14]. However, questions about the validity of the emotion measures in these studies have been raised, particularly the possibility that previous studies overlooked positive emotions that may be associated with cardiovascular responses. This is because most prior research primarily focused only on negative emotions. [6]. To address these research gaps, the current study investigated whether subjective emotions, including positive ones, are linked to cardiovascular responses to stressors.

Taken together, the study hypothesized that online and in-person conditions could demonstrate attenuated reactivity to stress compared with the alone condition. Additionally, as many studies demonstrated [6, 8, 14], if the presence of a friend exerts a direct effect on the cardiovascular system, no differences in subjective emotion would be observed based on the presence of friends during or post-stress. To observe the impact of these conditions as clearly as possible, the study controlled for the degree of closeness with friends.

## Materials and methods

### Participants

The study recruited 46 undergraduate students (Male = 21, Female = 24, blank = 1; age: 19–24 years, *M* = 20.54, *SD* = 1.21) via online advertisements through ********. They participated with a close friend (regardless of gender) and were paid 1,000 yen. After the experiment instructions were given, the participants provided written informed consent.

A minimum cell size of 15 was determined based on recent research using a similar paradigm [16]. Additionally, sample size estimation was conducted using G*Power v.3.1. A meta-analysis on the effects of social support on laboratory stressors (especially in speech delivery stress tasks) exhibited medium to large effect sizes [22]. Based on this, 45 participants were estimated to be sufficient to detect a 3-by-3 mixed ANOVA between-subjectseffect (*f* = .40, *α* = .05, 1-*β* = .80, number of groups = 3, and number of measurements = 3, were entered, respectively.) Our experiment aims to detect interaction effects in mixed-design ANOVA. However, we calculated the sample size based on the between-subjects design because it has been noted that G*Power drastically underestimates the sample size required to power an interaction effect [23].

The participants who failed to complete the questionnaire (*n* = 1) or expressed thoughts about the stress task as deception and took medication for high BP (*n* = 1) were excluded. The final sample consisted of 44 participants (Men: 19, Female: 24; average age: 20.52 years [*SD* = 1.21, range = 19–24 years]). In terms of distribution, 15, 14, and 15 participants were assigned under the in-person, online, and alone conditions, respectively.

### Measures

The study used the Two Dimensions Mood Scale (TDMS) to measure emotion and arousal [24, 25]. The TDMS is composed of eight items that measure emotional valence (negative-positive) and arousal levels (low–high). Items (e.g., “How energetic you are?”) were rated using a six-point scale (1: *not at all*, 6: *extremely*). The scores for emotional valence and arousal were calculated according to Sakairi et al. [24].

The study used BP and HR to examine differences in physiological responses to the presence of a friend. BP was evaluated using a cuff-sociometric semiautomatic device (OMRON HCR-7201). The participants were generally in a seated position, and the measurement of BP (SBP and DBP) was performed on the upper arm of the non-dominant hand. HR data were acquired using a Polar H10 heart rate sensor during the experiment, which fitted around the solar plexus. Data were transmitted via Bluetooth^®^ to an iPhone app (HR Variability Logger) [26, 27]. Autonomic activity was analyzed using the Kubios HRV Version 3.5 (Kubios Oy) using the RR interval of the ECG. Each record was previously analyzed to detect the potential presence of artifacts and anomalous beats by applying corresponding filters if required. Especially, the artifacts were checked using an iPhone app (HRV Logger) with data transmitted via Bluetooth^®^ as well as Kubios. Thus, HR that were significantly out of line, in addition to numerous artifacts, were preliminarily excluded from the raw data.

The HR variables were as follows: time domain analysis index of the RMSSD and frequency domain measurements, including low-frequency power (LF [ms^2^]), 0.04–0.15 Hz, high-frequency power (HF [ms^2^], 0.15–0.4 Hz), and LF/HF (ratio of absolute LF power to HF power). The LF in the LF power range obtained by frequency analysis of HR variability reflects sympathetic and parasympathetic nervous system activities, while the HF in the HF power is an index of parasympathetic nerve activity. Additionally, the LF/HF ratio is an index of sympathetic nerve activity. The LF/HF ratio is frequently used as a simple index of sympathetic nervous activity (or an index to evaluate the balance between the sympathetic and parasympathetic nervous systems).

Demographics, manipulation checks, and the relationship between the participants and friends were measured. For manipulation check, the study posed questions such as “During the experiment, how long was your friend in your sight?” and “During the experiment, how much did you feel like you were cooperating with your friend?” They were rated using a six-point Likert-type scale (1 = *not at all*, 6 = *very much*). For the relationship with a friend, the study posed the question: “How would you rate your friends who attended this experiment today?” with the following options: 1 = *best friends*, 2 = *up to about the third friend*, 3 = *about the third to fifth friend*, and 4 = *about the or less than the sixth friend*. This ranking was used as a continuous variable. Because even in the case of a Likert-type scale with unequal spacing across categories, using such data as continuous variables is still considered appropriate [28].

### Procedures

**Fig 1:**
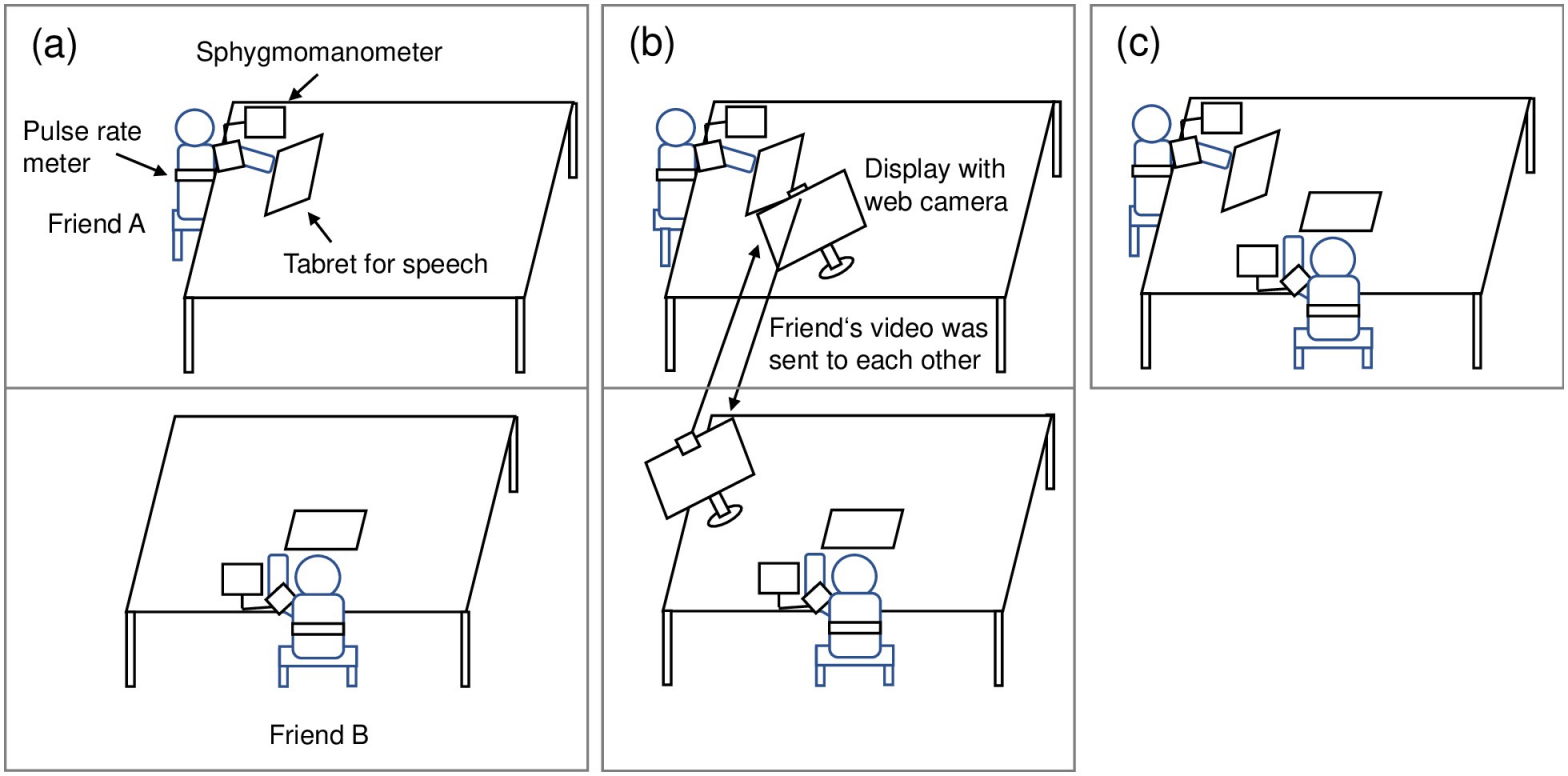
Experimental setup for each condition. (a) Alone condition. Participants were in a different room from their friends. (b) Online condition. Although the participants were in a different room from their friends, the two could see each other via web cameras. (c) In-person condition. Participants were in the same room.

The participants were separated into different rooms and instructed to sit in a chair (Fig 1). Before entering the room, the participants were informed that speaking to other participants was prohibited during the experiment. The researcher then entered the room and explained that measurements would be taken of their psychological and physiological states. They were provided with an informed consent form that described that the ethics committee of Kyoto University of Advanced Science (reference number: 21–523) approved the study, which was performed in accordance with the ethical guidelines of the American Psychological Association.

The participants returned the signed informed consent form. They were then equipped with an HR monitor and a sphygmomanometer and briefed on the use of the sphygmomanometer. Afterward, they practiced measuring BP using this device with instructions from the experimenter over the microphone.

This experiment was composed of three periods, namely, pre-, during, and post- (recovery) stress; cardiovascular responses to a stressor are characterized by increased activity during the stressful event followed by a decline toward pre-stress status [19]. In the pre-stress state, BP was measured (three times at one-time point) after a rest and a wait time of 3 min, and they completed the TDMS. During stress, the participants performed a speech preparation task in which they were presented with the names of six audience members on a tablet (Microsoft Surface). They were then instructed to give a speech using an online meeting system, in which they would describe their strengths and were given 3 min to prepare. BP was measured, and they completed the TDMS. After stress, the researcher apologized and told the participants that giving the speech was unnecessary. Once again, BP was measured after a wait time of 3 min, and they completed the TDMS.

To examine the effects, the presence of a friend was manipulated as alone, in-person, and online conditions. The participant and friend sat in separate rooms (alone and online conditions: Fig 1a and 1b) or in the same room (in-person condition: Fig 1c). In the online condition, a display screen and web camera were set on the desk, and the video feeds of the participant and friend were sent to each other. Under the online condition, their Zoom microphones were turned off from the beginning. In the in-person condition, their rooms were marked with a sign prohibiting conversation. At the end of the experiment, the participants completed the questionnaires, which included demographics, medications, and relationships with the friend. Afterward, they were fully debriefed.

## Results

### Data collection and analysis

Age and other manipulation checks were collected after the experiment (All data are available at S1 Dataset). In terms of the relationship between the participants and friends, the study found that the majority of the participants (88.6%) ranked the friend as *up to the fifth friend* (1: *N* = 10, 22.7%, 2: *N* = 19, 43.2%, 3: *N* = 10, 22.7%, 4: *N* = 5, 11.4%).

### Manipulation check

In terms of subjective experience, the participants under the in-person and online conditions reported that their friend had been in their sight more than those in the alone condition, *F*(2, 41) = 37.53, *p* < .001, *η*^2^ = .65. In contrast, the study observed no significant differences among the conditions in the scores for subjective experiences of collaboration, *F*(2, 41) = 0.78, *p* = .47, *η*^2^ = .04, which confirmed that the participants under the online and in-person conditions only felt the presence of their friend but did not feel collaborating with them.

One-way repeated measures ANOVAs with the factor of time points (pre-, during, and post-stress) revealed significant differences across the three-time points for positive affect, *F*(2, 86) = 21.84, *p* < .001, *η*^2^ = .34, and arousal affect, *F*(2, 82) = 23.27, *p* < .001, *η*^2^ = .36. For positive affect, there was a significant decrease from pre-stress to during-stress (*p* < .001). For arousal affect, there was a significant increase from pre-stress to during-stress (*p* < .001). This result confirmed that the stress task successfully elicited a psychological stress response.

### Condition and self-reported affect

Table 1 reports the means and standard deviations for self-reported affect (across the three time points and three conditions). The study found no significant differences among the three conditions at the pre-stress level (baseline) of the positive, *F*(2, 41) = 0.53, *p* = .59, and arousal, *F*(2, 41) = 0.14, *p* = .87, affects.

**Table 1:** Means of self-reported affect.

	Pre-stress	During-stress	Post-stress
Positive affect			
In-person	27.60	20.20	27.73
Online	26.64	24.79	27.79
Alone	25.93	22.07	27.40
Arousal			
In-person	11.00	13.93	10.67
Online	10.14	12.93	10.07
Alone	10.93	14.47	9.80

Prior to testing for statistical significance, the study calculated changes in the scores (change score) by subtracting the pre-stress score from the during/post-stress score to address the pre-stress score as baseline (zero). The change scores for positive and arousal affects were analyzed using a 3 (condition: in-person, online, and alone) × 2 (time point: during and post) mixed analysis design with the friend’s rank as a covariate. For positive affect, there was a significant main effect of time point, *F*(1, 40) = 19.03, *p* < .001, *η*^*2*^ = .11, which indicates that the participants felt less positive affect during stress than after stress. No significant main effect was observed for condition, *F*(2, 40) = 2.24, *p* = .12, *η*^2^ = .06, and no significant effect was found for interaction, *F*(2, 40) = 3.03, *p* = .06, *η*^2^ = .03.

For arousal affect, there was no significant main effect for time point, *F*(1, 40) = 3.20, *p* = .08, *η*^2^ = .02, and condition, *F*(2, 40) < 0.01, *p* > .99, *η*^2^ < .01. These main effects were not qualified by a significant tendency of interaction, *F*(2, 40) = 0.72, *p* = .49, *η*^2^ = .01.

### Condition and physiological responses

Table 2 presents the descriptive statistics of the physiological scores for the three time points for each condition. There were no significant differences among the three conditions in terms of the pre-stress levels (baseline) of HF, *F*(2, 41) = .83, *p* = .45; LF/HF, *F*(2, 41) = 1.49, *p* = .24; HR, *F*(2, 41) = 0.86, *p* = .43; SBP, *F*(2, 41) = 3.01, *p* = .06; and DBP, *F*(2, 41) = 2.49, *p* = .10.

**Table 2:** Descriptive statistics for physical responses.

	Pre-stress	During-stress	Post-stress
HF			
In-person	270.63	162.52	299.34
Online	249.17	176.58	219.82
Alone	203.82	156.64	182.14
LF/HF			
In-person	1.35	2.69	1.28
Online	1.67	1.94	1.56
Alone	2.75	2.47	2.24
RMSSD			
In-person	22.11	19.39	21.87
Online	19.20	17.88	19.19
Alone	20.20	17.76	20.04
HR			
In-person	80.41	95.70	79.58
Online	84.83	94.89	84.68
Alone	84.81	95.79	84.64
SBP			
In-person	101.09	110.04	100.84
Online	102.90	111.67	101.88
Alone	110.26	117.98	108.04
DBP			
In-person	67.29	73.80	67.29
Online	66.33	74.26	65.19
Alone	72.40	80.13	78.00

Prior to testing for statistical significance, the study calculated changes in the score of each physiological index by subtracting the pre-stress score from that during/post-stress to consider the pre-stress score as baseline (zero). Changes in the scores of the physiological responses were analyzed using a 3 (condition: in-person, online, and alone) × 2 (time points: during and post) mixed of analysis with the friend’s rank as the covariate. The significant main effects of time points appeared in HF, *F*(1, 40) = 5.80, *p* = .02, *η*^2^ = .03; HR, *F*(1, 40) = 12.78, *p* < .001, *η*^2^ = .12; and SBP, *F*(1, 40) = 6.76, *p* = .01, *η*^2^ = .05, but not in LF/HF, *F*(1, 40) = 1.77, *p* = .19, *η*^*2*^ = .02; DBP, *F*(1, 40) = 0.11, *p* = .74, *η*^*2*^ = .001; and RMSSD, *F*(1, 40) = 2.93, *p* = .09, *η*^*2*^ = .02. This result indicates that HF was significantly lower during stress compared with that post-stress and that HR and SBP were significantly higher during stress compared with that post-stress. None of the indices pointed to the significant main effect of the condition.

**Fig 2:**
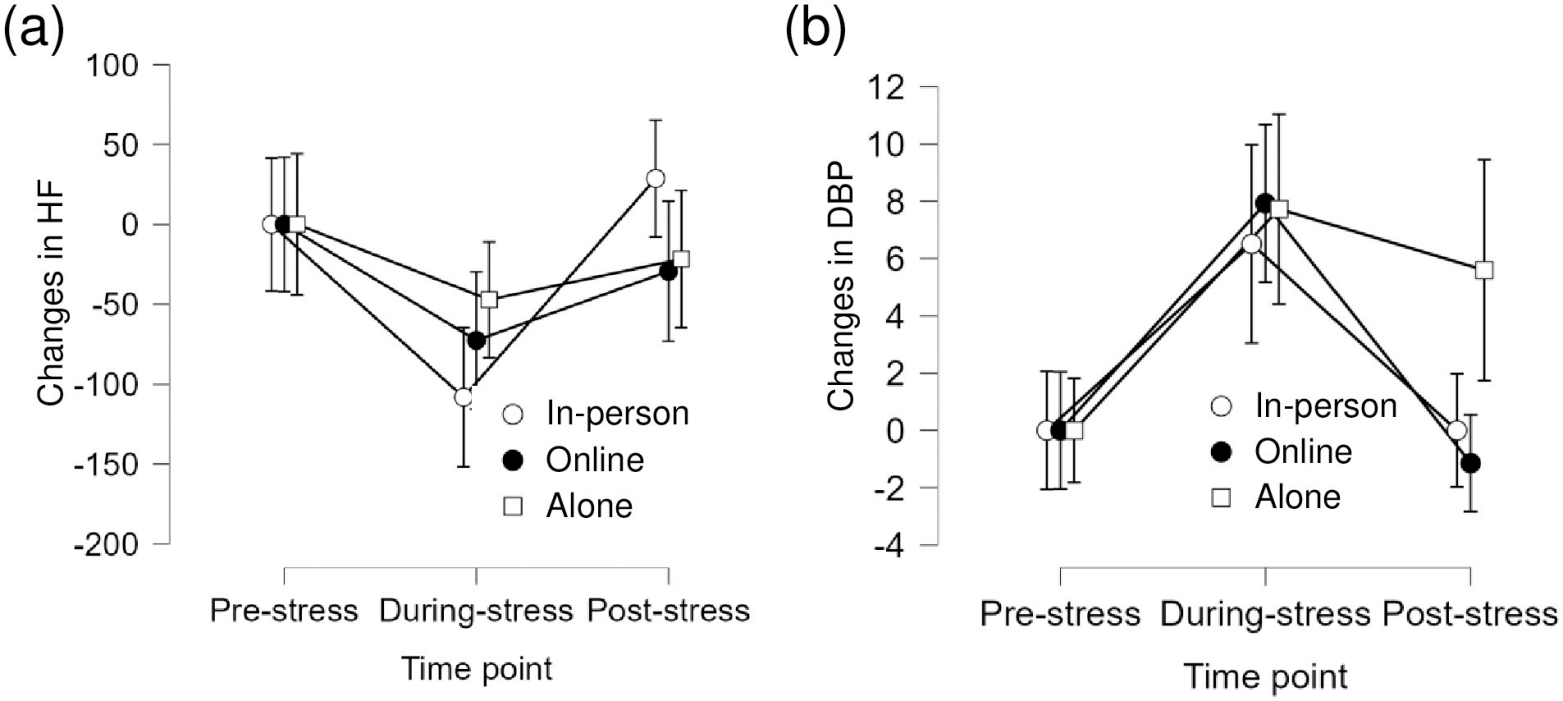
Changes in HF (a) and DBP (b). These values are the measured values minus the baseline (pre-stress) values. Error bars represent 95% confidence intervals.

The two-way interaction effects were significant in the change scores for HF, *F*(2, 40) = 5.10, *p* = .01, *η*^2^ = .05, and DBP, *F*(2, 40) = 3.56, *p* = .04, *η*^2^ = .07. Fig 2 presents the means and their 95% confidence intervals. The change score of HF under the in-person condition, the simple main effect of time points was significant, *F*(1, 14) = 27.29, *p* < .001, *η*^2^ = .31, which indicates that changes in the score for HF were significantly larger post-stress than that during stress under the in-person condition. This simple main effect of time points was nonsignificant for the online and alone conditions, *F*s < 2.28, *p*s > .16, *η*^*2*^s < .04. The other simple main effects and differences were nonsignificant.

Regarding changes in the scores for DBP, the simple main effect of time points was significant under the in-person and online conditions. These changes were significantly smaller at the post-stress than that during stress, *F*(1, 14) = 10.66, *p* = .006, *η*^2^ = .24; *F*(1, 13) = 34.45, *p* < .001, *η*^2^ = .45, respectively. The simple main effect of condition at the post-stress was also significant, *F*(2, 41) = 7.33, *p* = .002, *η2* = .26. At the post-stress, changes in the score for DBP were significantly smaller under the in-person and online conditions than that under the alone condition, *t*(41) = 3.00, *p* = .005; *t*(41) = 3.55, *p* = .001, respectively. The other simple main effects or differences were nonsignificant.

## Discussion

This study tested the hypothesis that the online and in-person conditions would display attenuated reactivity to stress compared with the alone condition, indicating less cardiovascular activation during stress and faster recovery to the original state after a certain period. The stress manipulation appeared effective, as evidenced by decreased positive affect in self-reported emotion during-stress compared to pre-stress levels.

For the main results, the effects of a friend’s presence on cardiovascular responses to stress were observed only in DBP out of the indicators, and this was only evident after stress. This result indicates that the presence of a friend does not reduce BP activation to stress but facilitates recovery after stress. Thus, the hypothesis was partially supported.

Possible reasons that no differences were observed in the scores for DBP during the stress task are as follows. DBP reflects BP due to mentally stressful situations perceived as beyond one’s control [20, 21]. Therefore, the presence of a friend did not influence the scores of DBP during the stress task, which is perceived as uncontrollable. Another interpretation is that anxiety due to the anticipation of evaluation from a friend may have caused additional psychological stress [29]. The stress-moderating effects of a significant other have not been observed when evaluation concerns were anticipated [10, 14, 20]. Studies demonstrated that friend’s presence reduces DBP during stress [9, 16], in which the participants did not expect that their friend (e.g., solving crossword puzzles while wearing headphones) would see the participants performing the task. In contrast, this study made the participants anticipated that their friends in the same room (in-person condition) or on a screen (online condition) would witness their performance (speech). Therefore, it is possible that while the presence of friends reduced stress, they also made the participants feel stressed due to evaluative concerns, resulting in no significant difference in DBP across the three conditions during stress task.

In this study, the effect of the friend’s presence was observed only in DBP but not in other indicators. Prior studies that examined the impact of the presence of friends also have not always demonstrated the effects in all measures, and shown inconsistent results across studies (e.g., HR and BP [8, 9, 12], only in SBP and DBP [13, 15], only in DBP [16], only in SBP [14]). The reasons for these discrepancies remain unclear [13]. However, Karmak et al. [13] posed that the buffering effect of a close other on cardiovascular responses clearly appears only when the stress task load is high. Although the stress task in the current study displayed a significant effect, its intensity was not relatively high (SBP increased by approximately 10) compared to tasks in prior studies. This cannot be conclusively determined from the current findings, however, the task load may be one of the reasons why the effect of the presence of a friend was not observed in the other cardiovascular indices.

The objective of the study was to examine whether or not the online or in-person presence of a friend have a stress-buffering effect compared with the alone condition. Thus, the objective was achieved by the observation that DBP was lower under the online and in-person conditions compared with under the alone condition. Nevertheless, it is noteworthy that the results of this study do not indicate that the effects of friends exert a general impact on cardiovascular indices.

For other indicators, the statistical results for HF and RMSSD indicated that parasympathetic activity differed in terms of the main effects of the time points. Especially under the in-person condition, the simple main effect of time point was significant, which implied that the change in HF was significantly larger after stress than that during stress in terms of the scores for HF. RMSSD is known to be less sensitive to respiratory rate [30] and is a widely used statistic when evaluating parasympathetic activity. The current study did not control for or measure respiration; thus, presenting a definite view on respiration is impossible. Therefore, the in-person condition may include a few effects on respiration. For this reason, future studies should include respiration as a variable and examine the factors that influence cardiovascular responses to the person at one’s side.

Finally, in the subjective emotion indices, the study found no differences in comfort and arousal between the three groups during or post-stress. Scholars have argued whether the effect of the presence of a supportive other on the cardiovascular system is mediated by subjective emotions or directly influenced by the cardiovascular system without subjective emotional changes [6, 8]. The present study supported the claim of a direct cardiovascular effect without intervening subjective emotional change.

In summary, the findings suggest that the mere presence of a friend, even merely online, may help unconsciously to recover BP to its level before stress from overactivity due to stress. DBP after stress was a better predictor of mild hypertension than that during stress [31]. Therefore, even if the presence of a friend exerted an effect only on DBP after stress, the result of this study suggested that spending time with close others online may improve health hazards due to isolation.

This study is the first to demonstrate the stress-buffering effect of the online presence of close others. However, it has certain limitations. The participants were relatively young and healthy. We should be cautious about those cardiovascular responses to stress may vary depending on age and health conditions. Particularly, participants are considered to belong to Generation Z, who grew up using online technologies to develop and maintain friendships during their adolescence [32, 33]. Given the fact that the effects of eating with others online on the autonomic nervous system were also observed in an older generation [18], this stress-buffering effect of the online presence of fiends may not only be limited to Generation Z. However, we should be cautious about whether the impact of the presence of friends through monitor can be generalized to other generations. Future studies will need to consider a larger sample size and a more diverse population. The second limitation is that the effect of the presence of a friend was not observed in the cardiovascular indices, except for DBP after stress. The potential causes for this result are evaluation concerns from friends and the level of the stress task load. Therefore, further investigation with various stress situation is required.

## Conclusions

We examined whether the stress-buffering effect of a friend could be observed even via online. The findings suggest that the online presence of a friend may help the recovery of BP from acute stress activity, similar to in-person situations without one’s awareness. Given that long-term isolation can lead to cardiovascular diseases, these findings indicate the importance of meeting with close others, even through a screen.

## Supporting information

S1 Dataset This is the anonymized data obtained in this experiment. (XLSX)
